# N-Doped HNT/TiO_2_ Nanocomposite by Electrospinning for Acetaminophen Degradation

**DOI:** 10.3390/membranes13020204

**Published:** 2023-02-07

**Authors:** Mahmoud Abid, Elissa Makhoul, Fida Tanos, Igor Iatsunskyi, Emerson Coy, Geoffroy Lesage, Marc Cretin, David Cornu, Abdesslem Ben Haj Amara, Mikhael Bechelany

**Affiliations:** 1 Institut Européen des Membranes, IEM, UMR 5635, Univ. Montpellier, ENSCM, CNRS, 34730 Montpellier, France; 2 Laboratory of Resources, Materials & Ecosystem (RME), Faculty of Sciences of Bizerte, University of Carthage, Zarzouna 7021, Tunisia; 3 NanoBioMedical Centre, Adam Mickiewicz University, Wszechnicy Piastowskiej 3, 61-614 Poznan, Poland

**Keywords:** halloysite nanotubes, TiO_2_ nanofibers, electrospinning, nitriding, photocatalysis, acetaminophen

## Abstract

In this study, we combined electrospinning of a large amount of halloysite (HNT, 95%) with nitriding to produce N-HNT-TiO_2_ composite nanofibers (N-H95T5 hereafter) to be used for acetaminophen (ACT) photodegradation. Investigation of the morphological and structural properties of the obtained materials did not highlight any significant difference in their morphological features and confirmed that nitrogen was evenly distributed in the samples. Photocatalytic tests under visible light showed that acetaminophen photodegraded faster in the presence of samples with nitrogen (N-H95T5) than without (H95T5 nanofibers). Moreover, the N-H95T5 nanocomposite photocatalytic activity did not change after repeated utilization (five cycles). The addition of scavengers during photocatalytic tests showed the key implication of OH^•−^, O_2_^•−^ and h^+^ radicals in acetaminophen degradation. These results indicated that N–H95T5 composite nanofibers could be considered a cheap multifunctional material for photodegradation and could open new prospects for preparing tunable photocatalysts.

## 1. Introduction

Water pollution is a major problem leading to health and environmental issues [1]. With proper water treatment, such as by heterogeneous photocatalysis, it is possible to reduce water pollution by degrading pollutants [2,3,4]. TiO_2_ is the most used material in water purification systems because of its low cost, low toxicity, relatively high efficiency, and high chemical and thermal stability [5,6]. However, two of its major drawbacks are the limited recovery after water treatment and the rapid recombination of the photogenerated charges [7]. Therefore, substantial research has been conducted to optimize TiO_2_ photocatalytic activity [8,9]. For instance, doping and surface modification have been studied to improve the utilization of sunlight. TiO_2_ has been doped using metal ions (e.g., Pd [10,11], Ag [12,13], Pt [10]), non-metal ions (e.g., B, N, Cu, Ni), and semi-conductors (e.g., BN, ZnO, CuO) [14,15,16,17] to enhance its photocatalytic activity.

According to the atomic orbital theory, nitrogen doping increases the amount of nitrogen in the first energy level N 1s and in the O 2p orbital of TiO_2_. This allows for increasing the number of electrons in the conduction band and the number of holes in the valence band and limiting the in situ recombination of electron-hole pairs after excitation with visible light [18]. In nitrogen-doped TiO_2_, the semiconductor bandgap is reduced (thus facilitating the photocatalyst activation), the light response range is broadened, and the number of photogenerated carriers is increased [19,20]. Recently, Asahi et al. showed that TiO_2_ optical absorbance into the visible light region is enhanced after nitrogen doping [21]. Nitrogen-doped TiO_2_ (N-TiO_2_) can be prepared using different methods, such as hydrothermal synthesis and calcination in the presence of NH_3_ [22] and by post-synthesis nitriding at 500 °C. In the last study, the authors highlighted the improved activity of N-TiO_2_ for acetone oxidation under visible light [23]. Moreover, nitrogen doping by treatment with ammonium nitrate in water at pH 5.87 strongly increased N-TiO_2_-mediated degradation of 2,4-dichlorophenol following exposure to visible light [24]. These findings indicate that nitrogen doping improves TiO_2_ photocatalytic activity by promoting hydroxyl and superoxide radical production.

Recently, Abid et al. prepared TiO_2_-based composite nanofibers with 95% halloysite (H95T5) and demonstrated that these nanocomposites degraded 91% of acetaminophen under visible light irradiation in 360 min [25]. Here, we explored the preparation and photocatalytic performance of nitrogen-doped H95T5 (N-H95T5) composite nanofibers. We found that these new nitrogen-doped nanocomposites eliminated more than 95% of acetaminophen in the presence of visible light in only 270 min. We then used high-performance liquid chromatography (HPLC) and toxicity assays to determine the intermediates and reaction products generated during the photocatalytic process and their toxicity and found out that the percentage of bacterial luminescence inhibition by ACT decreased to 6% after 24 h using N-H95T5 compared to 52% with H95T5 [25]. We identified the main reactive species explaining acetaminophen degradation using scavenger tests.

## 2. Materials and Methods

### 2.1. Materials

All chemicals listed in Table 1 were used without further purification.

### 2.2. Preparation of N-H95T5 Composite Nanofibers

N-H95T5 samples were prepared by electrospinning and nitriding following previously described protocols [25,26]. Polyvinylpyrrolidone solution was prepared by dissolving PVP powder into ethanol solution. Then, halloysite was added to TiO_2_ prepared by hydrolyzing titanium tetraisopropoxide into a mixture of acetic acid and ethanol. The precursor mixture was stirred for 1 h at room temperature and electrospun. The obtained nanofibers were left exposed to air overnight to hydrolyze followed by heating at 400 °C for 4 h in air and sintering in a tubular furnace at 500 °C for 1 h (under nitrogen atmosphere).

### 2.3. Structural Characterizations

The samples’ structures, phase, and crystallinity were determined by scanning electron microscopy (SEM; Hitachi S-4800) and transmission electron microscopy (TEM; JEOL ARM 200F). Surface and micropore areas were described using the Brunauer-Emmett-Teller (BET) method, with different data points and relative pressures (P/Po) from 0 to 1. Their structural and crystallinity properties, elemental composition, and oxidation states were analyzed by X-ray diffraction (XRD; PANAlytical Xpert-PRO diffractometer equipped with an X’celerator detector using Ni-filtered Cu-radiation), Fourier-transform infrared spectroscopy (FT-IR) was recorded with the NEXUS instrument, equipped with an attenuated total reflection accessory in the frequency range of 400–4000 cm^−1^, Raman (Horiba Xplora, 532 nm), and X-ray photoelectron spectroscopy (XPS) with an ESCALAB 250 spectrometer (Thermo Electron; excitation source: Al Kα monochromatic source, 1486.6 eV), respectively.

### 2.4. Electrochemical Activity

Electrochemical impedance spectroscopy was performed as previously published [25,27].

### 2.5. Photocatalytic Activity

For photocatalytic activity testing, acetaminophen degradation in the presence of different samples and of visible light was carried out as in our previous work [25] and quantified with Equation (1) [28].

Degradation efficiency (%) = [(C_0_ − C)/C_0_] × 100, (1)

where C_0_ and C are the pollutant concentrations before and after irradiation.

Acetaminophen was chosen for these tests because it is consumed in all countries of the world, has been detected in water samples from different origins, and is very stable in water [14,17,25,29,30,31,32].

### 2.6. Kinetics

Acetaminophen photocatalytic degradation kinetic data were fitted using a pseudo-first-order kinetic model [33]:

ln (C_0_/C) = K_app_ t, (2)

where C_0_ is the initial concentration, C is the concentration at time t, and K_app_ is the apparent rate constant.

### 2.7. Micro-Toxicity Tests

To assess the toxicity of the solution during acetaminophen photodegradation, micro-toxicity tests were carried out using the bioluminescent marine bacterium *Vibrio fischeri* as described in [25].

## 3. Results and Discussion

### 3.1. H95T5 and N-H95T5 Morphology and Structure

H95T5 and N-H95T5 morphological features were characterized by SEM. In both samples, nanofibers were uniform, continuous, and randomly oriented (Figure S1a,b). This confirmed that the introduction of nitrogen did not change TiO_2_ shape, in good agreement with the literature [22,25,34].

Crystallinity analysis of H95T5 and N-H95T5 by XRD showed (Figure S2) the characteristic 001 reflection of HNT (7.18 Å) at 2θ, and TiO_2_ anatase phase with tetragonal arrangement reflections at nearly 2θ = 25.36, 37.7, 48.06, 54.01, 55.00, 62.49, 68.61, 69.68, and 74.85° ascribed to the (101), (112), (200), (105), (211), (204), (116), (220), and (215) Miller plans, respectively [25]. XRD patterns indicated that the main TiO_2_ reflection was shifted from the initial position after nitriding. Likewise, no significant difference was found between H95T5 and N-H95T5 by Raman and Fourier-transform infrared spectroscopy (Figure S3).

**Figure 1:**
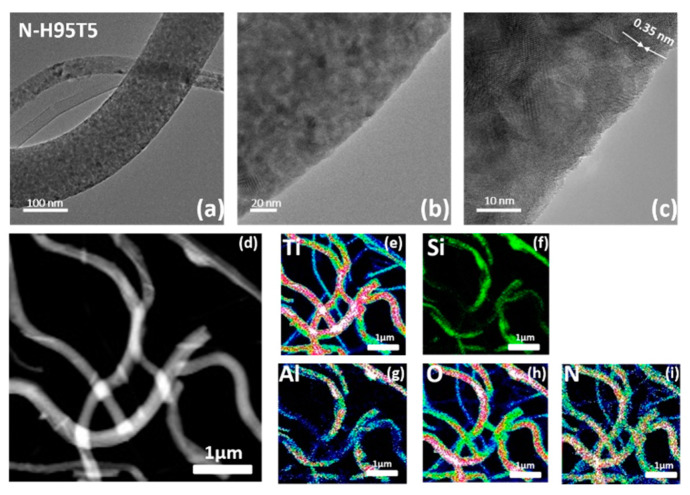
TEM images (**a**–**c**) and STEM-EDX chemical mapping of N-H95T5 (**d**–**i**).

High-resolution TEM (Figure 1a–c) indicated that N-H95T5 nanofibers had a rough and large surface area with a lattice spacing of 0.350 nm, fully consistent with the distance of the (101) crystalline plane of the TiO_2_ anatase [25,35,36]. Doping with nitrogen changed neither the morphology of N-H95T5 compared with H95T5 [25] nor the crystal lattice values (selected area electron diffraction images). In fact, elemental mapping can be used for qualitative (the type of elements) as well as quantitative (the percentage of the concentration of each element of the sample) analysis. In N-H95T5, there are more than four elements. Then, it is recommended to proceed with the quantitative analysis by indicating the concentration of elements by a change in color: from blue (low concentration) to red (high concentration) to green in the middle. Figure 1d–i confirmed the homogenous distribution of nitrogen with T, O, Al, and Si elements.

This section may be divided by subheadings. It should provide a concise and precise description of the experimental results, their interpretation, as well as the experimental conclusions that can be drawn.

**Figure 2:**
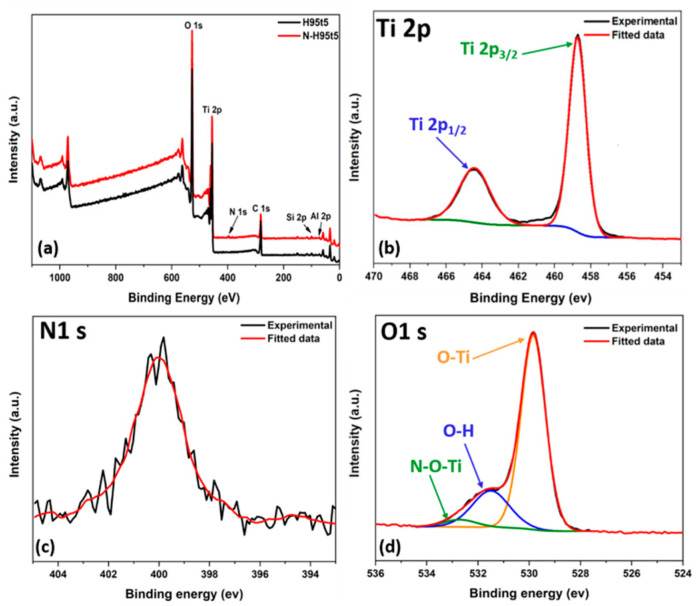
Comparison of the X-ray photoelectron spectra of H95T5 and N-H95T5 (**a**) and high-resolution XPS spectra of (**b**) Ti 2p, (**c**) N 1s, and (**d**) O 1s.

N-H95T5 surface structure and chemical state were investigated by XPS. The survey spectrum of H95T5 and N-H95T5 (Figure 2a) contained the dominant signals of Ti 2p and O 1s and the weak signals of C 1s, Al 2p, and Si 2p. In comparison, nitrogen content can be detected in the nitrogen-treated H95T5 sample. Figure S4 shows the N 1s peak for these samples before and after nitriding. For further analysis of the chemical structure of the N-H95T5 samples, high-resolution spectrum of XPS was used to identify the elements present in the N-H95T5 nanofibers in three areas. The Ti 2p region near 460 eV (Figure 2b), the N 1s region near 400 eV (Figure 2c), and the O 1s region near 530 eV (Figure 2d).

The Ti 2p spectrum (Figure 2b) presented two peaks (458.73 and 464.43 eV: Ti 2p_3/2_ and Ti 2p_1/2_). After deconvolution of the N 1s peak, only one peak was detected with binding energy at 399.82 eV (Figure 2c). The N 1s binding energy peak was broad, ranging from 396.32 eV to 403.91 eV and centered at 399.82 eV, which was greater than the typical binding energy of 397.2 eV in Ti-N; therefore, Ti-N bonds were excluded and this peak could be assigned to the presence of O-Ti-N linkage [37,38]. In the O 1s spectrum (Figure 2d), the peaks at 529.84 and 531.50 eV were assigned to O-Ti and O-H. respectively. The XPS spectra displayed a third peak that shifted to a higher binding energy (+0.7 eV) after nitriding (Table 2). This bond could be attributed to the Ti-O-N or O-Ti-N bonds mainly on the surface [39,40]. The XPS spectra confirmed nitrogen distribution into H95T5 nanofibers.

**Figure 3:**
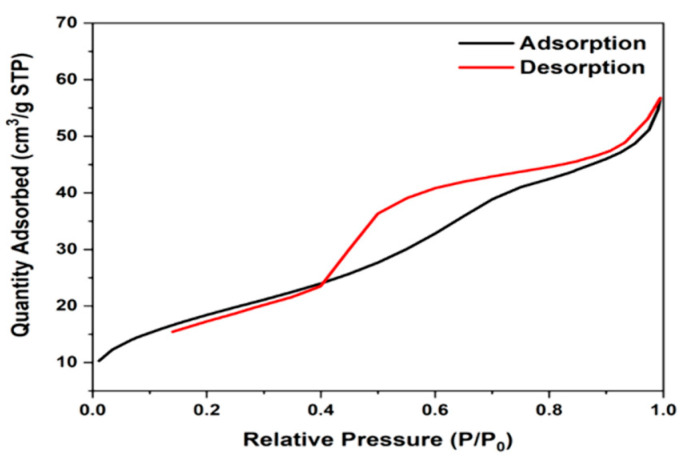
Nitrogen adsorption-desorption isotherms of N-H95T5.

Nitrogen is commonly used for BET surface analysis because of its high purity and strong interaction with most solids. Since the interaction between gas and solid phases is generally weak, the surface is cooled with liquid N_2_ to obtain detectable levels of adsorption. A known amount of nitrogen gas is then gradually released into the sample cell. Relative pressures less than atmospheric pressure are achieved by creating conditions of partial vacuum. After the adsorption layer is formed, the sample is removed from the nitrogen atmosphere and heated at room temperature to release the adsorbed nitrogen from the material and quantify it. Heating from −200 °C to 25 °C has no great effect on the surface morphology and architecture of N-H95T5 nanofibers. Figure 3 demonstrates that the N-H95T5 exhibited a type IV isotherm and a type H2 hysteresis loop at a lower relative pressure region [41]. The BET method gave specific surface area values of 36.6 m^2^/g for H95T5 and 67.85 m^2^/g for N-H95T5. This increase was due to nitrogen incorporation in TiO_2_ nanofibers and should be beneficial for photocatalytic activity by creating more active adsorption sites [42].

### 3.2. Electrochemical Activity

**Figure 4:**
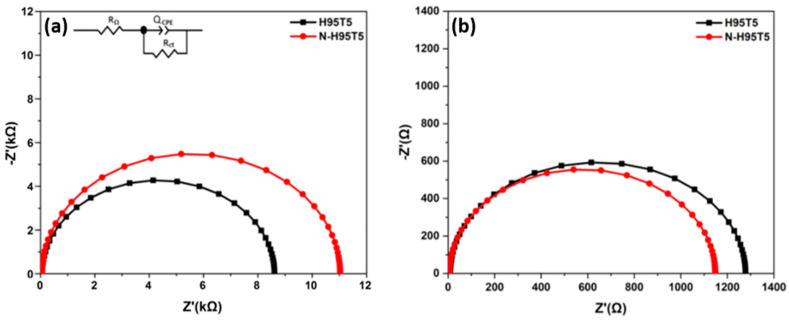
Nyquist curves of H95T5 and N-H95T5 in the dark (**a**) and with visible light (**b**). The proposed circuit is shown in the inset.

Electrochemical impedance spectroscopy with the previously described model was used to investigate N-H95T5 electrochemical activity [25]. Figure 4a,b show that the impedance arc radius of electrodes in the dark was much bigger than that under visible light irradiation, which indicated that there were few electrons across the electrolyte interfaces in the dark. While under visible light, the arc radius of the N-doped H95T5 electrode was smaller than that of the undoped electrode. This demonstrated that the N-H95T5 displayed greater separation efficiency of photogenerated electron-hole pairs and faster charge transfer than H95T5. It can be seen that the resistance of N-H95t5 decreased by 89% after its exposure to visible light irradiation compared with 85% for H95T5. Charge transfer rate was 10% faster and photogenerated electron-hole separation improved, as indicated by the lower R_2_ value (1148 Ω for N-H95T5 versus 1280 Ω for H95T5). This indicates that heat treatment with an inert nitrogen atmosphere is a promising way to improve the efficiency of photocatalyst.

**Figure 5:**
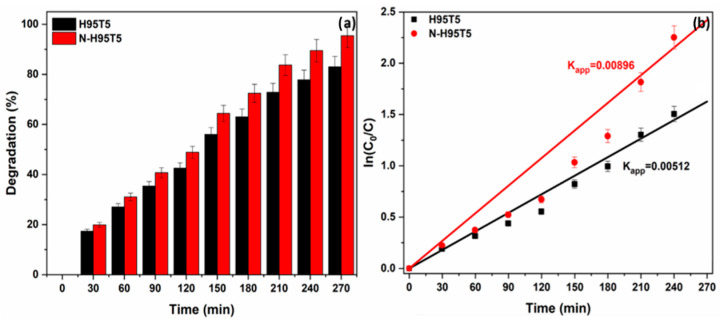
Degradation rate of acetaminophen in the presence of H95T5 and N-H95T5 upon exposure to visible light (**a**) and acetaminophen degradation kinetics (**b**). Data are the mean value of three measurements and the relative error is lower than ±5%.

Then, acetaminophen degradation quantification in the presence of N-H95T5 or H95T5 and visible light for 4.5 h (Figure 5a) showed an increase in photocatalytic efficiency with N-H95T5 at the end of the experiment (83% and 95% of acetaminophen degraded with H95T5 and with N-H95T5, respectively). These data suggest that nitrogen doping increases oxygen vacancies that promote the trapping of photoinduced electrons and that act as a reactive center for photocatalysis [23,43].

Table 3 summarizes the degradation activity of previously studied photocatalysts for organic pollutants in water, highlighting the comparable performance of N-H95T5.

Acetaminophen degradation kinetics was explained by a pseudo first-order reaction (curve linearity and linear coefficient *R*^2^~1) (Figure 5b). The lower electron-hole recombination rate explained the enhanced acetaminophen degradation with N-H95T5.

After the photocatalysis experiment, the photocatalyst was removed by filtration, washed with deionized water several times, and dried at 80 °C for 12 h, then treated for 15 min at 500 °C to remove all impurities and water molecules. Then, the catalyst was analyzed by BET. The BET method gave specific surface area values of 678,512 m^2^/g for N-H95T5 and 60.8532 m^2^/g for regenerated N-H95T5. A loss of 11% was found but the obtained value remained higher than raw H95T5.

**Figure 6:**
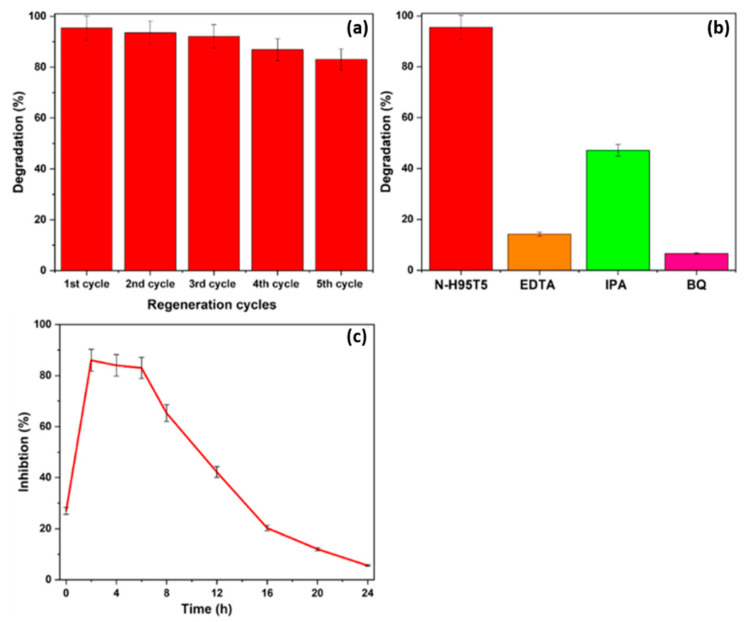
N-H95T5 is a stable photocatalyst (**a**). Acetaminophen photodegradation in the presence of N-H95T5 and of the indicated scavengers (**b**). Vibrio fischeri fluorescence inhibition during acetaminophen degradation by N-H95T5 (**c**). IPA, isopropanol; BQ, p-benzoquinone.

N-H95T5 reusability was confirmed by monitoring acetaminophen (10 mg/L, pH 7) degradation over five cycles (same conditions as before). After each experiment, N-H95T5 was filtered, washed in water, and dried (100 °C for 12 h). Acetaminophen degradation reached 95.45% in the first run and then was 93.54%, 92.11%, 86.95%, and 83.04% (Figure 6a). The decrease of 17% after five consecutive cycles could be related to nitrogen loss after each run or to the accumulation of degradation by-products on the catalyst surface that decrease the number of available active sites [48]. Despite this loss of activity after five cycles, N-H95T5 can be considered a promising stable catalyst for industrial applications.

Then, acetaminophen degradation assays were performed using the same experimental conditions, but in the presence of N-H95T5 and different scavengers (0.06M [49]) to determine what reactive radicals are implicated in this photocatalytic process [49] (Figure 6b). The addition of isopropanol (^•^OH scavenger) strongly decreased the acetaminophen degradation rate. The strongest inhibitory effects were obtained with benzoquinone (O_2_^•−^ scavenger) and with EDTA (h^+^ scavenger). Therefore, all three radical types are implicated in acetaminophen photodegradation [17,25].

Lastly, toxicity assays were performed to monitor the formation of harmful by-products during acetaminophen degradation. When *Vibrio fischeri* was incubated with N-H95T5 and acetaminophen, its natural fluorescence was inhibited by 27% after 15 min and up to 86% after 2 h of exposure to visible light to induce acetaminophen degradation (Figure 6c). Fluorescence inhibition progressively decreased: 83% at 6 h, 65% at 8 h, 42% at 12 h, 12% at 20 h, and 6% at 24 h. This indicates that after 24 h, the toxic aromatic by-products generated during acetaminophen photodegradation were transformed into nontoxic compounds [33,50,51].

## 4. Conclusions

This study describes the fabrication of H95T5 and N-H95T5 composite nanofibers by combining electrospinning and nitriding. After doping with nitrogen, no significant difference was found between H95T5 and N-H95T5 by Raman, FT-IR, XRD, and SEM. In addition, nitrogen was homogeneously distributed with T, O, Al, and Si as proved by elemental mapping. In the XPS survey spectrum, Ti 2p and O 1s were the dominant signals, while C 1s, Al 2p, Si 2p, and N 1s were weaker. The charge transfer rate was faster and electron-hole separation was improved by nitrogen doping. Nitrogen doping also enhanced the catalytic properties of the prepared sample, with a degradation rate of 0.0089 min^−1^. Recyclability tests were promising, as indicated by the loss of only 17% of activity after five cycles. The scavenging experiments revealed that ^•^OH, h^+^ and O_2_^•−^ were strongly implicated in acetaminophen photodegradation. Toxicity (i.e., *V. fischeri* fluorescence inhibition) was high in the first 4h of acetaminophen photodegradation in the presence of N-H95T5, due to the generation of aromatic by-products (1,4-benzoquinone, benzoic acid, and benzaldehyde) that were later transformed into nontoxic compounds. This study has confirmed that a cheap photocatalyst with low TiO_2_ and nitrogen concentrations can be used for the degradation of organic molecules and has opened prospects for mass production and practical applications.

## Figures and Tables

**Table 1:** Materials used in this work, suppliers, mass fraction purity, and CAS number.

Material	Supplier/Source	Purity	CAS Number
Halloysite (HNT)	Tamra (Nefza District, NW Tunisia)	-	-
Titanium tetraisopropoxide (TTIP)	Sigma–Aldrich	97%	546-68-9
Polyvinyl pyrrolidone (PVP)	Sigma–Aldrich	Mw = 1,300,000	9003-39-8
Sodium chloride	Sigma–Aldrich	≥99%	7647-14-5
Silver nitrate	Sigma–Aldrich	≥99%	7761-88-8
Acetaminophen (ACT)	Sigma–Aldrich	≥99%	103-90-2
2-propanol (IPA)	Sigma–Aldrich	99.9%,	67-63-0
p-benzoquinone (BQ)	Sigma–Aldrich	≥99.5%,	106-51-4
Ethylenediaminetetraacetic acid (EDTA)	Sigma–Aldrich	99.995%,	60-00-4
Acetic acid	VWR chemicals	-	64-19-7
Ethanol	VWR chemicals	≥99.8%,	64-17-5
Deionized water	Milli-Q^®^ Academic	>18.2 MΩ	

**Table 2:** Deconvoluted peaks of O 1s and Ti 2p.

H95T5	Position	N-H95T5	Position
O 1s O-Ti	529.7	O 1s N-O-Ti	529.84
O 1s O-H O=C	531.1	O 1s O-H	531.50
O 1s O-Si O-C	532.0	O 1s O-Ti	532.70
Ti 2p_3/2_ TiO_2_	458.5	Ti 2p_3/2_ TiO_2_	458.73
Ti 2p_1/2_ TiO_2_	464.2	Ti 2p_1/2_ TiO_2_	464.43 eV

**Table 3:** Photocatalytic activity of different photocatalysts.

Pollutant (mg/L)	Photocatalyst (g/L)	Synthesis Technique	Visible Light Source	Degradation Efficiency (%)	Degradation Time (min)	Ref.
Acetaminophen (ACT) (10 mg/L)	N-H95T5 (0.5 g/L)	Electrospinning + nitriding	Halogen linear lamp	95	270	This work
ACT (10 mg/L)	H95T5 (0.5 g/L)	Sol-gel + electrospinning	Halogen linear lamp	91	360	[25]
Rhodamine B (10 mg/L)	N-TiO_2_ (1g/L)	Sol-gel + ammonia treatment	500 W mercury lamp	90	120	[22]
Gaseous toluene	N–TiO_2_	Sol-gel + ammonia atmosphere treatment	150 W Xe lamp with an IR cutter	48	60	[21]
Rhodamine B (20 mg/L)	N-TiO_2_ (1g/L)	Microemulsion− hydrothermal method	1000 W halogen lamp	96	60	[44]
2,4-dichlorophenol (2,4-DCP) (100 mg/L)				56	300	
Methyl Orange (MO) (20 mg/L)	N-TiO_2_-400 (0.062 g/L)	Precipitation + ammonium hydroxide	Visible light	52	836	[45]
2,4-DCP (100 mg/L)	N-TiO_2_ (1g/L)	Sol-gel+ NH_4_NO_3_/NH_3_ H_2_O	1000 W halogen lamp	52	300	[24]
ACT (5 mg/L)	N-TiO_2_-NTs (0.5g/L)	ALD + nitriding	Halogen linear lamp	98	90	[34]
MO (9.8 mg/L)	N-TiO_2_ (1g/L)	Solid state dispersion + urea	Solar light	91	90	[46]
Methylene Blue (MB) (10 mg/L)	N-TiO_2_ (1g/L)	Wet chemical method + urea	Visible light	72	180	[47]

## Data Availability

Not applicable.

## Supplementary Materials

The following supporting information can be downloaded at: https://www.mdpi.com/article/10.3390/membranes13020204/s1, Figure S1: SEM micrographs showing H95T5 (a) and N-H95T5 nanocomposite fibers (b); Figure S2: XRD patterns of H95T5 and N-H95T5 nanocomposite fibers; Figure S3: FT-IR spectra (a) and Raman spectra (b) of H95T5 and N-H95T5 nanofibers; Figure S4: XPS survey spectra of H95T5 and N-H95T5 from 420 to 380 ev.
