# Supplementary material for: N-Doped HNT/TiO2 Nanocomposite by Electrospinning for Acetaminophen Degradation
Source: Membranes (Basel). 2023 Feb 7;13(2):204. doi: 10.3390/membranes13020204 (PMC9967914; doi:10.3390/membranes13020204)
Supplement: Supplementary file 1 [file membranes-13-00204-s001.zip › membranes-2138823-supplementary.pdf]

Supporting information

N-Doped HNT/TiO_2 Nanocomposite by Electrospinning for Acetaminophen Degradation

Mahmoud Abid [1,2], Elissa Makhoul [1], Fida Tanos [1], Igor Iatsunskyi [3], Emerson Coy [3], Geoffroy Lesage [1], Marc Cretin [1], David Cornu [1,*], Abdesslem Ben Haj Amara [1,†] and Mikhael Bechelany [1,*,†]

[1] Institut Européen des Membranes, IEM, UMR 5635, Univ Montpellier, ENSCM, CNRS, 34730 Montpellier, France
[2] Laboratory of Resources, Materials & Ecosystem (RME), Faculty of Sciences of Bizerte, University of Carthage, Zarzouna 7021, Tunisia
[3] NanoBioMedical Centre, Adam Mickiewicz University, Wszechnicy Piastowskiej 3, 61-614 Poznan, Poland
* Correspondence: david.cornu@umontpellier.fr (D.C.); mikhael.bechelany@umontpellier.fr (M.B.)
† These authors contributed equally to this work.

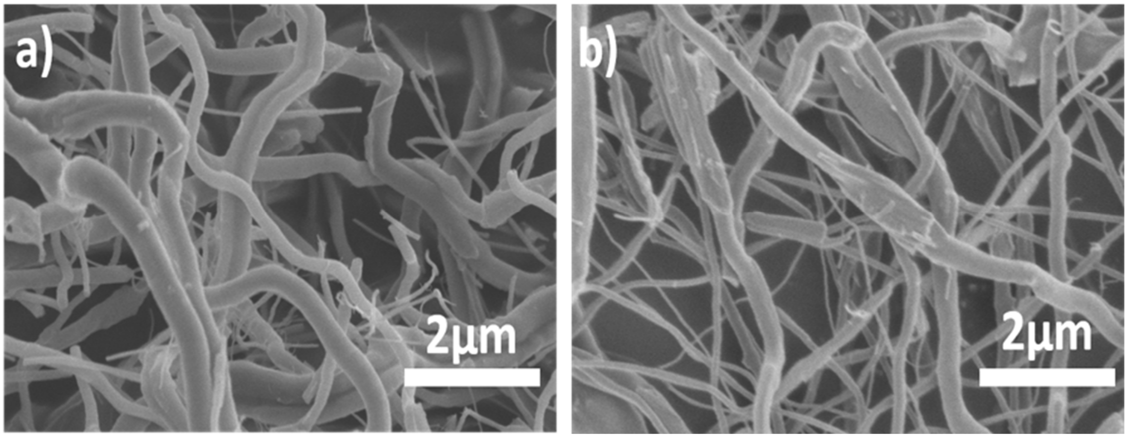

Figure S1. SEM micrographs showing H95T5 (a) and N-H95T5 nanocomposite fibers (b).

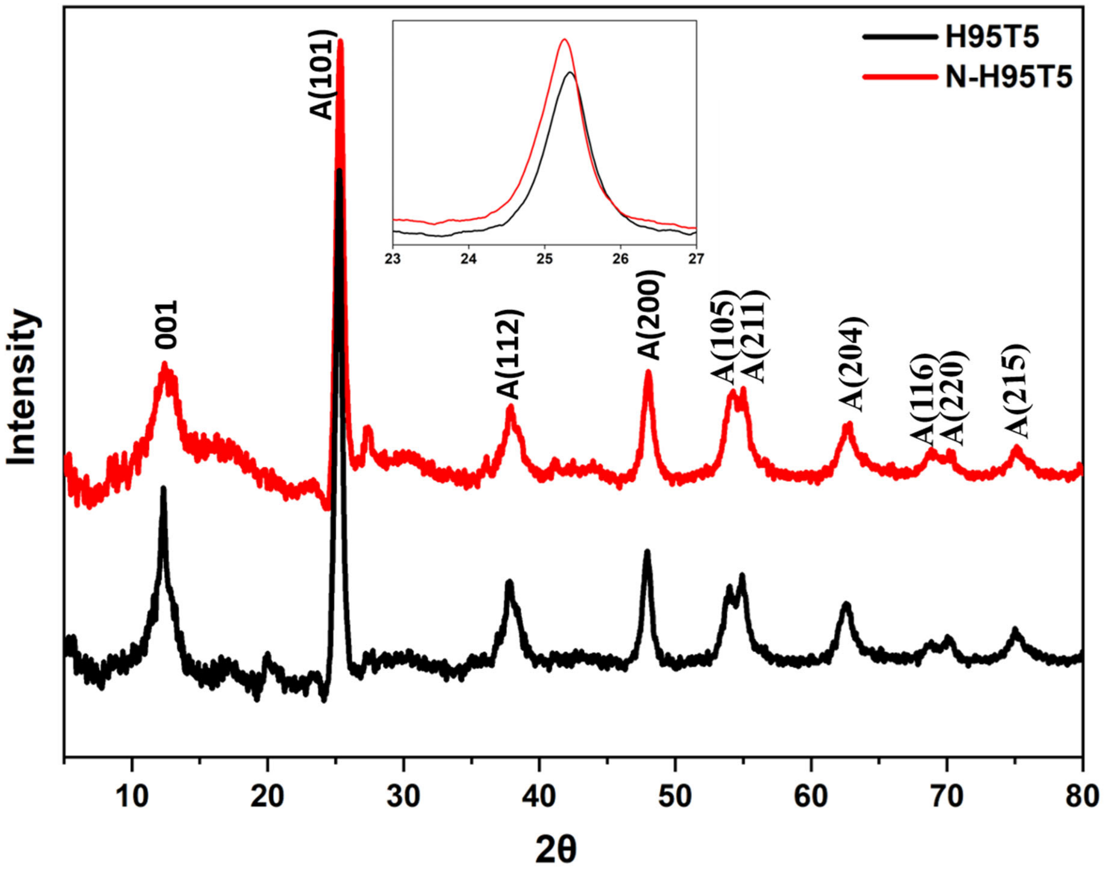

Figure S2. XRD patterns of H95T5 and N-H95T5 nanocomposite fibers.

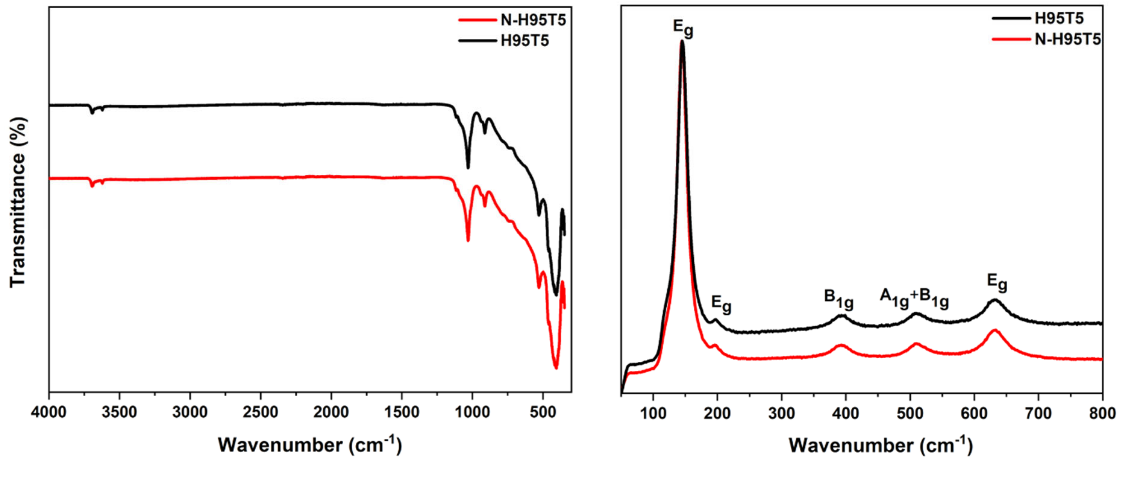

Figure S3. FT-IR spectra (a) and Raman spectra (b) of H95T5 and N-H95T5 nanofibers

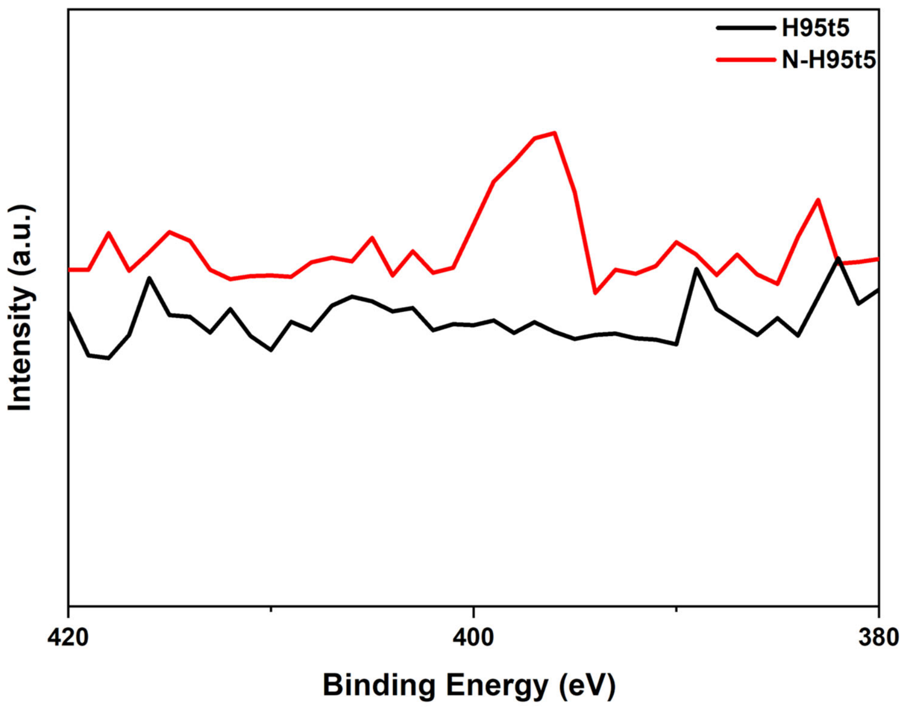

Figure S4. XPS survey spectra of H95T5 and N-H95T5 from 420 to 380 eV
